# Reduction of routine use of radiography in patients with ankle fractures leads to lower costs and has no impact on clinical outcome: an economic evaluation

**DOI:** 10.1186/s12913-020-05725-1

**Published:** 2020-09-22

**Authors:** P. van Gerven, J. M. van Dongen, S. M. Rubinstein, M. F. Termaat, M. El Moumni, W. P. Zuidema, P. Krijnen, I. B. Schipper, M. W. van Tulder, on behalf of the WARRIOR study group, L. van Bodegom-Vos, R. S. Breederveld, R. J. Derksen, B. van Dijkman, J. C. Goslings, J. H. Hegeman, J. M. Hoogendoorn, C. van Kuijk, S. A. G. Meylaerts, F. R. Rosendaal, N. L. Weil, K. W. Wendt

**Affiliations:** 1 Department of Traumasurgery, Leiden University Medical Center, P.O. Box 9600, 2300 RC Leiden, the Netherlands; 2 Department of Health Sciences, Faculty of Science, Amsterdam Movement Sciences research institute, Vrije Universiteit, Amsterdam, the Netherlands; 3 Department of Surgery, University of Groningen, University Medical Center Groningen, Groningen, the Netherlands; 4 Department of Surgery, Amsterdam University Medical Centers, Amsterdam, the Netherlands; 5 Department of Physiotherapy and Occupational Therapy, Aarhus University Hospital, Aarhus, Denmark

**Keywords:** Cost-effectiveness analysis, Economic evaluation, Radiography, Randomised controlled trial, Ankle fractures, Routine, Follow-up

## Abstract

**Background:**

To evaluate the cost-effectiveness of a reduction in the number of routine radiographs in the follow-up of patients with ankle fractures.

**Methods:**

We performed an economic evaluation alongside the multicentre, randomised WARRIOR trial. Participants were randomised to a reduced imaging follow-up protocol (i.e. radiographs at week 6 and 12 follow-up obtained on clinical indication) or usual care (i.e. routine radiography at weeks 6 and 12). The Olerud & Molander Ankle Score (OMAS) was used to assess ankle function and the EQ-5D-3L was used to estimate Quality-Adjusted Life Years (QALYs). Costs and resource use were assessed using self-reported questionnaires and medical records, and analysed from a societal perspective. Multiple imputation was used for missing data, and data were analysed using seemingly unrelated regression analysis and bootstrapping.

**Results:**

In total, 246 patients had data available for analysis (reduced imaging = 118; usual care = 128). Fewer radiographs were obtained in the reduced imaging group (median = 4) compared with the usual-care group (median = 5). Functional outcome was comparable in both groups. The difference in QALYs was − 0.008 (95% CI:-0.06 to 0.04) and the difference in OMAS was 0.73 (95% CI:-5.29 to 6.76). Imaging costs were lower in the reduced imaging group (−€48; 95% CI:- €72 to -€25). All other cost categories did not statistically differ between the groups. The probability of the reduced imaging protocol being cost-effectiveness was 0.45 at a wiliness-to-pay of €20,000 per QALY.

**Conclusions:**

Reducing the number of routine follow-up radiographs has a low probability of being cost-effective compared with usual care. Functional outcome, health-related quality of life and societal costs were comparable in both groups, whereas imaging costs were marginally lower in the reduced imaging group. Given this, adherence to a reduced imaging follow-up protocol for those with routine ankle fractures can be followed without sacrificing quality of care, and may result in reduced costs.

**Trial registration:**

The trial was registered on 26-05-2014 in the Netherlands Trial Registry, with reference number NL4477 (www.trialregister.nl/trial/4477).

## Background

Ankle fractures are common and account for about 9% of all fractures in the UK [1]. The incidence of ankle fractures around the world is reported to lie between 71 and 187 per 100,000 persons per year and has risen over the last decade due to ageing of the population and increased participation in athletic activities [2–5]. Routine imaging during the follow-up of ankle fractures is associated with relatively high healthcare costs [6, 7]. Healthcare costs are expected to rise in coming decades [8]. This has led to an increased interest in the effectiveness of imaging in clinical decision-making [9–12]. Despite increased costs, both national and international trauma protocols dictate that routine radiographs should be obtained at regular intervals during the follow-up of patients with an ankle fracture, although there is little scientific evidence to support this position [4, 13, 14]. For both non-operatively and operatively treated patients, it is recommended that four outpatient clinic visits including radiographs, are to be conducted after a follow-up of one, two, six and twelve weeks [13]. The goal of these radiographs are to monitor the position of the fracture fragments, the position of fixation material, the alignment of the joint and the bone-healing process.

In the Netherlands, with a population of over 17 million, approximately €5 million is spent annually on radiography for patients with ankle fractures. This estimate is based on an incidence of 30,000 per annum [15], with three to four follow-up radiographs [16], at a cost of €50 per radiograph [17]. Various studies have questioned the value of routine radiographs obtained at the first outpatient clinic visit and at intermediate-to-late follow-up (i.e. after the initial 3 weeks) of operatively treated ankle fractures [18, 19]. A recent retrospective analysis, involving a cohort of 528 patients with an ankle fracture, demonstrated that as few as 1.2% (*n* = 11/928) of routine radiographs obtained after 3 weeks of follow-up led to a change in treatment strategy [16]. These results suggest that current imaging protocols for the follow-up of ankle fracture patients might not be cost-effective.

## Methods

### Aim

The aim of this study was to evaluate the cost-effectiveness of a protocol with reduced numbers of routine radiographs in the follow-up of ankle fractures, in comparison with the current usual care.

### Design

This economic evaluation was conducted alongside a multicentre, randomised controlled trial. The methods of this trial, including its sample size calculation, are described in detail elsewhere [20]. Both a cost-effectiveness and cost-utility analysis were performed from a societal perspective. The time horizon of the economic evaluation was 12 months. Consolidated Health Economic Evaluation Reporting Standards (CHEERS) guidelines were followed in preparing this report [21, 22].

### Setting

Seven hospitals in the Netherlands participated in the study, including three university hospitals and four large teaching hospitals. Patients were enrolled between July 2014 and October 2017.

### Participants

#### Inclusion/exclusion criteria

Patients could participate in the study if they provided written informed consent, were over 18 years of age, had a fracture of the ankle (Lauge Hansen classification types Supination Adduction [SA]2, Supination External rotation [SE]2–4, Pronation External rotation [PE] 1–4 or Pronation Abduction [PA] 1–3) [23] and were able to independently complete the Dutch questionnaires. Distortions or isolated Danis-Weber type A fractures [24] were not included. Exclusion criteria were the presence of fractures to multiple extremities and pathologic or open fractures (Gustillo grades 2–3). If patients were deemed unable to comply with follow-up they were also excluded.

#### Randomisation

Patients were informed about the study both verbally and by means of an information letter. All participants had to provide written informed consent. Participants were randomised by means of computerised allocation, using an online registration and randomisation program (ProMISe; Project Manager Internet Server; https://www.msbi.nl/promise/ProMISe.aspx). Participants were assigned in a 1:1 ratio to either the intervention group or the control group. Randomisation was carried out using a stratified, randomly varying block design (each block size containing 2 to 6 allocations). The tables were internally pre-generated within the secure data management system and stratified by hospital and initial treatment strategy.

#### Control group – usual care

Patients randomised to the usual care group were monitored at the outpatient clinic and received routine follow-up radiographs at one, two, six and twelve weeks of follow-up. Additional follow-up moments with or without the use of radiographs could be scheduled at any time if deemed necessary by the treating physician.

#### Intervention group – reduced imaging

In the reduced imaging group, radiographs were routinely obtained after one and two weeks. Radiographs could be obtained later during follow-up if a specific clinical indication was present or obtained at the discretion of the treating physician. Reasons for doing so included an additional trauma to the affected ankle, a pain score of 6 or higher on a 11-point Numerical Rating Scale (NRS), a decrease in Range-Of-Motion (ROM), or neurovascular abnormalities. Motivations to obtain additional radiographs were required to be logged in the medical file. Aside from the modified imaging protocol at follow-up, all aspects of treatment and follow-up were similar for both groups.

### Outcome measures

Measurements at baseline included potential confounders [25], such as age, sex, medical history, smoking habits, alcohol intake, functional status, health-related quality of life (HR-QOL) and socioeconomic status. Follow-up questionnaires assessing the patients’ clinical outcomes as well as their resource use were administered after 6, 12, 26, and 52 weeks and could be completed either online or by post. Recall periods of these questionnaires varied from 6 weeks at 6-week follow-up to 26 weeks at 52-week follow-up to cover the complete duration of follow-up.

#### Clinical outcomes

Functional status of the affected ankle was evaluated using the Olerud and Molander Ankle Score (OMAS). This is a nine-item questionnaire assessing both pain and disability related to the affected ankle. OMAS scores were calculated for all of the measurement points separately, ranging from 0 to 100 with a score of 100 equalling no pain or disability [26]. HR-QOL was assessed using the Dutch version of the EQ-5D-3L. At baseline, participants were asked to complete the EQ-5D-3L for their health state just prior to their trauma. At all other time points, they were asked to consider their current health status. Utility scores per time point were estimated using the Dutch EQ-5D-3L tariff [27, 28]. Quality-adjusted Life Years (QALYs) per patient were estimated using linear interpolation of the utility scores for the different time points. Since the patients’ utility score right after the trauma was not available (i.e. the patients’ “true” baseline utility score), we assumed their utility score at baseline to be equal to that of 6 weeks of follow-up.

#### Cost measures

Resource use questionnaires were used to measure the patients’ use of primary and secondary healthcare, medication, informal care, as well as their levels of unpaid productivity losses, absenteeism and presenteeism. Costs of the intervention (i.e. costs for the radiographs) were gathered from electronic patient records. Primary healthcare use included the patients’ number of general practitioner consultations, visits to a company medical officer, physiotherapy treatments and visits to other specialised therapists. All of these visits were required to be associated with the ankle fracture. Information on the use of secondary healthcare services was gathered from electronic patient records and included admissions to hospital, nursing home or rehabilitation centre, outpatient clinic visits, all imaging other than plain radiographs (e.g. CT- or MRI-scans of the ankle) and re-operations. These services also included the initial admission right after the trauma occurred, and the primary intervention, if applicable. All healthcare costs were valued according to Dutch standard costs [29] or, if unavailable, tariffs. Medication costs were calculated as costs-per-day for each medication, which was based upon the standard dosage per day and unit prices of the Royal Dutch Society of Pharmacy [30]. Total medication costs were calculated by multiplying this cost per day with the total days of use. If the duration was not specified, we assumed patients used a certain medication during the complete recall period. Unpaid productivity losses (i.e. volunteer work, caregiving or domestic activities patients could not perform due to their trauma) and informal care (i.e. care provided by relatives, friends or volunteers) were valued at €14.13 per hour, a shadow price that is recommended by the Dutch National Health Care Institute [29]. Absenteeism was defined as the number of days of absence due to the ankle fracture. The Friction Cost Approach was used to value absenteeism, which assumes that costs are limited to the time it takes to replace an absent worker (in the Netherlands: 12 weeks) [29]. The participants’ number of presenteeism days were estimated by multiplying the number of days worked (i.e. workable days – sickness absence days) by a self-reported score reflecting their productivity level when they were present at work ranging from 0 (equalling no productivity) to 10 (equalling full productivity). Days of absenteeism and presenteeism were valued using gender-specific price weights [29]. The trial’s follow-up was 12 months and discounting of costs and effects was, therefore, not necessary. All costs were converted to Euros 2016 with the help of consumer price indices [31].

### Statistical analysis

Analyses were performed in accordance with the intention-to-treat principle. Missing data were multiply imputed using STATA (Version 12 SE, Stata Corp, College station, TX). The imputation model included utility scores, OMAS scores, and all available cost values at baseline, 6, 12, 26 and 52 weeks as well as the baseline variables: fracture classification, BMI, ASA classification, smoking habits, alcohol intake, hospital, age, sex, randomisation result and operative- or non-operative treatment. These baseline variables were added because they were regarded as possible confounders, because they differed between groups at baseline, and/or because they were predictive of the ‘missingness’ of data. Five complete datasets were generated in order for the loss-of-efficiency to be lower than 5% [32]. Each dataset was analysed separately and estimates were pooled using Rubin’s rules. This method takes into account both imputation variability within each dataset, as well as imputation variability between the separate datasets [32]. Seemingly unrelated regression analyses (SUR) were used to estimate total cost (ΔC) and effect differences (ΔE). The advantage of SUR is that ΔC and ΔE are modelled simultaneously so that their possible correlation can be accounted for [33]. For OMAS, the patients’ 52-week follow-up scores were used as dependent variable. For total costs and QALYs, the patients’ total costs and QALYs experienced during follow-up were used as dependent variable, respectively. OMAS analyses were adjusted for baseline scores and other possible confounders measured at baseline (Table 1). In contrast to the recommendation of Manca et al. [34], we decided not to adjust QALYs for baseline utility scores, as a “true” utility score was lacking in the current study. That is, the baseline utility value in this study described the patients’ utility value prior to their fracture, instead of right after their fracture. The incremental cost-effectiveness ratio (ICER) was estimated by dividing the cost difference by the effect difference (ΔC/ΔE). To estimate the uncertainty around the ICER and to estimate 95% confidence intervals (95% CI) surrounding the cost differences, bias-corrected and accelerated bootstrapping was performed with 5000 replications. For all 5000 replications, the cost and effect pairs were plotted on a cost-effectiveness plane to graphically illustrate the uncertainty surrounding the ICER [35]. A summary measure of the joint uncertainty surrounding costs and effects was provided by constructing cost-effectiveness acceptability curves (CEACs). These curves give an indication of the probability that the reduced imaging protocol for ankle fractures is cost-effective for a range of willingness-to-pay values. CEACs were pooled using a combination of Rubin’s rules and the incremental net monetary benefit approach. Statistical significance was assumed at *p* < 0.05.

**Table 1:** Patient characteristics by treatment allocation

		usual care	reduced imaging
		(N = 128)	(*N* = 118)
**Male sex**, n (%)		69 (53.9)	58 (48.7)
**Age,** mean (SD)		47.7 (18.5)	50.8 (18.2)
**BMI,** mean (SD)		25.8 (4.3)	27.3 (6.0)
**Alcohol > 10 U/week,** n (%)		22 (17.2)	16 (13.4)
**Smoking > 10/day,** n (%)		10 (7.8)	9 (7.6)
**Operative treatment,** n (%)		77 (60.2)	77 (64.7)
**Lauge-Hansen**^**18**^ **classification,** n (%)	**SA**	2 (1.6)	2 (1.7)
	**SE**	94 (73.4)	94 (79.0)
	**PA/PE**	31 (24.2)	23 (19.3)
	**missing**	1 (0.8)	0 (0.0)
**Weber**^**19**^ **classification,** n (%)	**A**	2 (1.6)	2 (1.7)
	**B**	93 (72.7)	94 (79.0)
	**C**	27 (21.1)	21 (17.6)
	**missing**	6 (4.7)	2 (1.7)
**Malleolar involvement,** n (%)	**Uni-**	66 (51.6)	64 (53.8)
	**Bi-**	27 (21.1)	21 (17.6)
	**Tri-**	35 (27.3)	34 (28.6)
**ASA classification,** n (%)	**1**	53 (41.4)	47 (39.5)
	**2**	60 (46.9)	55 (46.2)
	**≥3**	15 (11.7)	12 (7.7)

Abbreviations: *SD* Standard deviation; *SA* Supination-adduction; *SE* Supination-external rotation; *PA* Pronation-adduction; *PE* Pronation-eversion; *BMI* Body Mass index; *ASA* American Society of Anesthesiologists, available from: www.asahq.org

#### Sensitivity analyses

A total of six sensitivity analyses were planned. In the first sensitivity analysis, only data of participants with complete data were used (SA1). The second sensitivity analysis (SA2) made use of the measured utility score at baseline (prior to the fracture), instead of the value derived from the utility score at 6 weeks. The third sensitivity analysis (SA3) used the Human Capital Approach to calculate productivity losses instead of the Friction Cost Approach. The Human Capital Approach assumes that productivity losses occur during the complete period of absence instead of being limited to the friction period. For the fourth sensitivity analysis (SA4), costs were assessed from a healthcare perspective. A healthcare perspective regards only costs accruing to the formal Dutch healthcare system, meaning that costs of informal care, absenteeism, presenteeism and unpaid productivity losses were disregarded. The fifth (SA5) and sixth sensitivity analysis (SA6) only included patients with either a non-operative or an operative treatment strategy, respectively.

## Results

### Participants

**Fig. 1:**
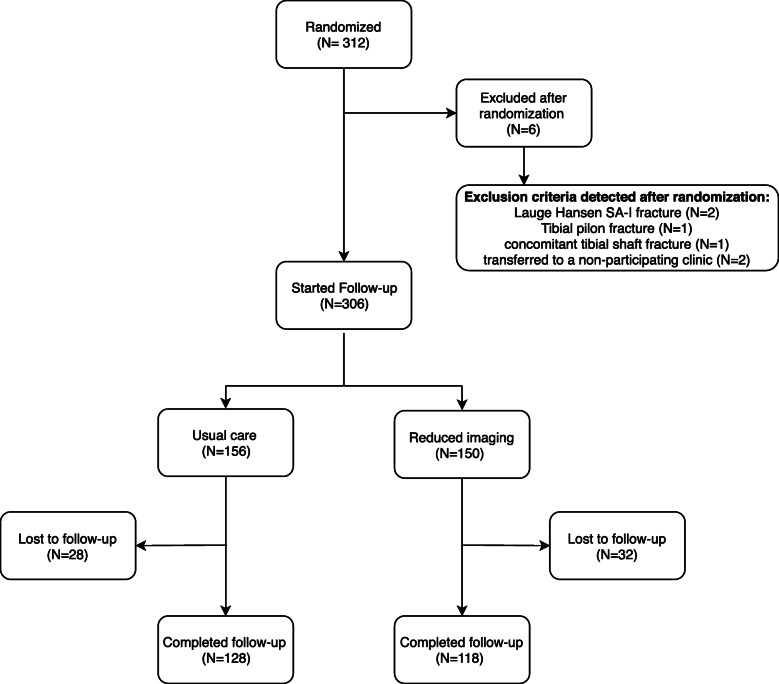
Flowchart of participants

We enrolled 312 participants in the study (Fig. 1). Six were excluded after randomisation, because an exclusion criterion was present that was not identified before randomization (Fig. 1). Of the remaining 306 participants, 156 were randomised to usual care and 150 to reduced imaging. In total, 60 patients, 28 in the usual care group (18%) and 32 in the reduced imaging group (21%) did not return any of the follow-up questionnaires and were lost to follow-up. Thus, 246 participants were included in the analysis (*n* = 128 usual care and *n* = 118 reduced imaging). Aside from a higher mean Body-Mass-Index (BMI) in the reduced imaging group, no meaningful differences were observed between groups at baseline (Table 1). Surgery was performed in 60% of participants in the usual care group (*n* = 77/128) and in 65% of participants in the reduced imaging group (*n* = 77/118). Out of a total of 1230 (246*5) baseline and follow-up questionnaires, 1096 were returned by the participants (89%).

### Effects

There was no statistically significant difference in OMAS (0.73; 95% CI:-5.3 to 6.8) and QALYs (− 0.008; 95% CI:-0.04 to 0.03) between groups. An overview of the patients’ OMAS and EQ-5D-3L scores per time point can be found in Additional file 1.

### Costs and use of resources

As a result of the intervention, patients randomised to the reduced imaging group had fewer radiographs taken of their ankle fracture than patients randomised to usual care, equalling a median number of radiographs of 4 (Interquartile Range [IQR] 3–5) in the reduced imaging group versus a median of 5 (IQR 4–6) in the usual care group. This resulted in a significant reduction in radiograph costs in favour of the reduced imaging group (−€48 per patient, 95% CI:-72 to − 25). All other costs, including total societal costs, were not statistically significantly different between groups (Table 2).

**Table 2:** Mean cost (in euros) per participant in the intervention and control group and mean cost differences between groups during follow-up

Cost category	Control *n* = 128, mean (SEM)	Intervention *n* = 118, mean (SEM)	Cost difference crude, mean (95%CI)	Cost difference adjusted, mean (95%CI)
Intervention	266 (9)	222 (9)	**−44 (−70 to − 20)**	**−48 (−72 to − 25)**
Primary care	967 (154)	1266 (387)	299 (− 170 to 1421)	137 (− 277 to 1018)
Secondary care	7435 (971)	7803 (1176)	368 (− 2156 to 3457)	− 169 (− 2230 to 2178)
Medication	36 (9)	27 (7)	−9 (−27 to 11)	−8 (− 27 to 12)
Informal care	671 (121)	647 (131)	−24(− 338 to 312)	−46 (− 373 to 262)
Absenteeism	976 (212)	1218 (312)	242 (− 397 to 1008)	306 (−373 to 1109)
Presenteeism	4903 (627)	4373 (605)	− 530 (− 2084 to 979)	−29(− 1503 to 1408)
Unpaid productivity loss	789 (152)	757 (184)	−32 (−467 to 410)	−12(− 437 to 427)
**Total**	**16,046 (1419)**	**16,314 (1741)**	**267 (− 3370 to 4778)**	**130 (− 2975 to 3723)**

### Cost-effectiveness

**Fig. 2:**
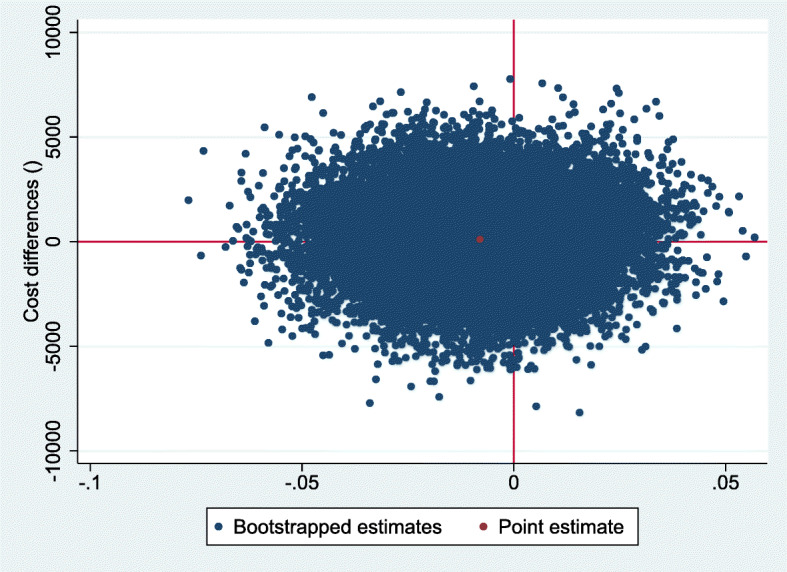
Cost-effectiveness plane for QALYs, representing the results from the 5000 bootstrapped replications, and the point estimate. Higher on the Y-axis corresponds to costlier than control, more right on the X axis corresponds to more effective than control

**Fig. 3:**
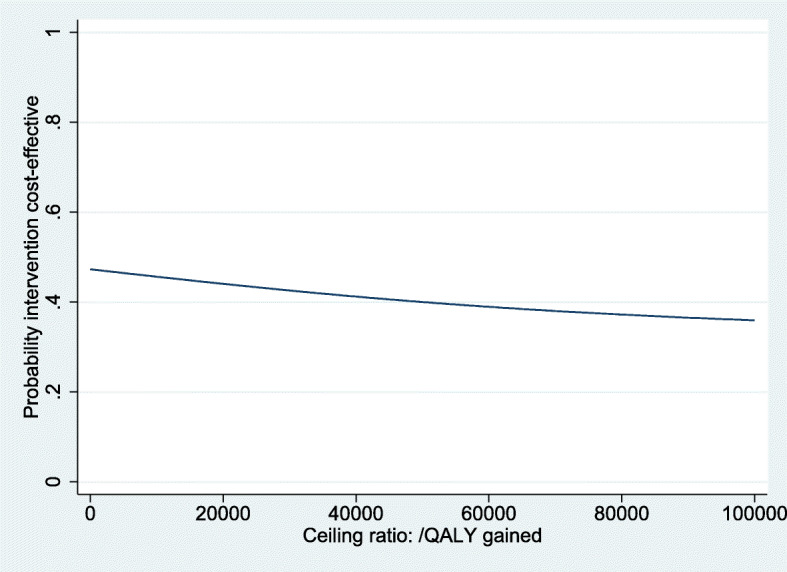
Cost-effectiveness acceptability curve for QALYs, showing the probability of the intervention being cost effective at a certain willingness-to-pay value per QALY

**Fig. 4:**
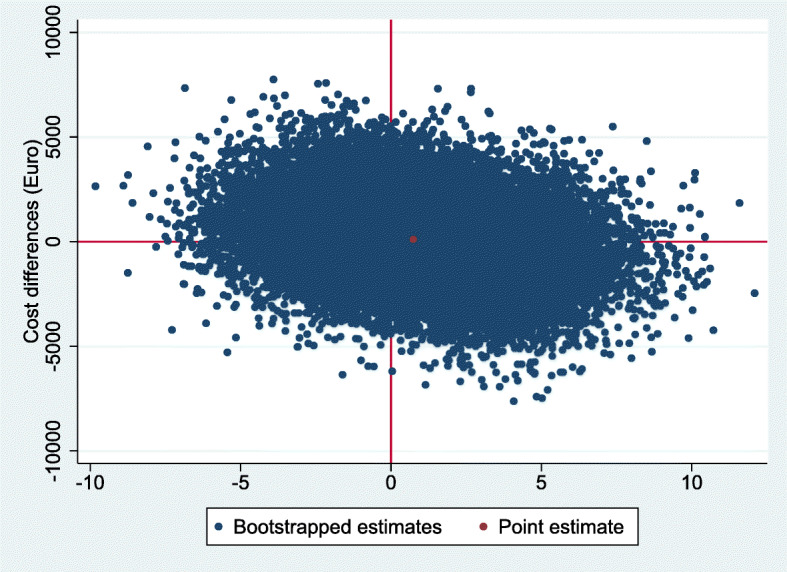
Cost-effectiveness plane for OMAS, representing the results from the 5000 bootstrapped replications, and the point estimate. Higher on the Y-axis corresponds to costlier than control, more right on the X axis corresponds to more effective than control

**Fig. 5:**
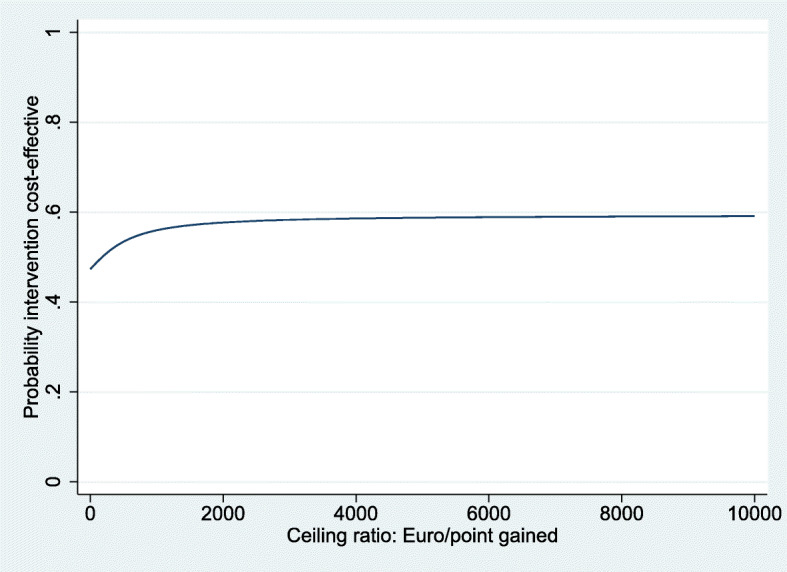
Cost-effectiveness acceptability curve for OMAS, showing the probability of the intervention being cost effective at a certain willingness-to-pay value per point increase of the OMAS

For QALYs, the intervention was dominated by the control, based on a cost difference (ΔC) of €131 and an effect difference (ΔE) of − 0.008 QALY. The ICER for functional outcome was 178, based on the same ΔC of €131 and a ΔE of 0.73 points on the OMAS (Table 3). The CE-plane for QALYs shows that the cost-effect pairs were scattered across all four quadrants of the CE-plane (Fig. 2). The CEAC in Fig. 3 indicates that if decision-makers are willing to pay €20,000 per QALY gained, the probability of reduced imaging being cost-effectiveness compared with usual care was 0.45. This probability reduced with increasing values of willingness to pay to about 0.37 at a willingness to pay of €80,000 per QALY. The CE-plane for the OMAS also shows that the cost-effect pairs were scattered across all four quadrants of the CE-plane (Fig. 4). For OMAS, the CEAC indicates that if decision-makers are not willing to pay anything per point improvement, the probability of reduced imaging being cost-effectiveness compared with usual care was 0.47. This probability increased with increasing values of willingness-to-pay to about 0.59 at a willingness to pay of €5000 per point improvement (Fig. 5).

**Table 3:** Differences in pooled mean costs and effects (95% Confidence intervals), incremental cost-effectiveness ratios, and the distribution of incremental cost-effect pairs around the quadrants of the cost-effectiveness planes for reduced imaging compared to usual care

Analysis	Sample size		Outcome measure	∆C (95% CI)	∆E (95% CI)	ICER	Distribution CE-plane (%)			
	Control	Intervention		€	Points	€/point	NE	SE	SW	NW
**Main analysis** - Imputed dataset	128	118	QALYs (Range: 0–1)	131 (− 3039 to 3928)	− 0.008 (− 0.04 to 0.03)	−16,198	17.4	14.4	32.1	36.0
	128	118	OMAS (Range: 0–100)	131 (− 3039 to 3928)	0.73 (− 5.29 to 6.76)	178	21.4	31.8	15.0	25.8
**SA1** – Complete cases	29	23	QALYs (Range: 0–1)	1242 (− 7949 to 6447)	−0.014 (− 0.06 to 0.04)	−86,988	46.0	7.5	10.9	35.6
	29	23	OMAS (Range: 0–100)	1242 (−7949 to 6447)	3.04 (− 5.82 to 11.89)	409	70.1	16.2	2.8	10.8
**SA2** – QALY 1 VS QALY 2	128	118	QALYs (Range: 0–1)	131 (−3039 to 3947)	−0.013 (− 0.05 to 0.02)	− 10,394	8.3	15.9	30.8	45.0
**SA3** – Human capital approach	128	118	QALYs (Range: 0–1)	383 (− 2900 to 4365)	−0.008 (− 0.04 to 0.03)	−47,311	19.2	13.2	29.8	39.9
	128	118	OMAS (Range: 0–100)	383 (− 2900 to 4365)	0.73 (− 5.29 to 6.75)	527	30.6	28.9	13.2	27.4
**SA4** – Healthcare perspective	128	118	QALYs (Range: 0–1)	− 89 (− 2386 to 3287)	−0.008 (− 0.04 to 0.03)	11,052	14.3	17.2	36.4	32.1
	128	118	OMAS (Range: 0–100)	−89 (−2386 to 3287)	0.73 (−5.29 to 6.75)	−121	23.6	36.2	17.4	22.8
**SA5 –** Conservative treatment	51	41	QALYs (Range: 0–1)	− 2425 (− 9691 to 1223)	−0.03 (− 0.09 to 0.02)	74,883	3.7	8.1	66.2	22.0
	51	41	OMAS (Range: 0–100)	−2425 (− 9691 to 1223)	− 1.60 (−10.49 to 7.30)	1519	6.2	28.5	45.5	19.8
**SA6 –** Operative treatment	77	77	QALYs (Range: 0–1)	1432 (− 3007 to 6706)	0.001 (−0.04 to 0.04)	1,504,404	39.0	15.5	14.7	30.9
	77	77	OMAS (Range: 0–100)	1432 (−3007 to 6706)	1.51 (−5.07 to 8.08)	951	50.6	24.7	4.3	20.4

*SA* Sensitivity analysis; *QALYs* Quality Adjusted Life Years; *OMAS* Olerud and Molander Ankle Score; *∆C* Difference in cost; *∆E* Difference in effect; *ICER* Incremental Cost Effectiveness Ratio; *CE- plane* Cost Effectiveness plane; *NE* North east part of the CE-plane (representing an intervention that is more costly, but more effective); *SE* South east part of the CE-plane (representing an intervention that is cheaper, and more effective); *SW* South west part of the CE-plane (representing an intervention that is cheaper, but less effective); *NW* North west part of the CE-plane (representing an intervention that is both more costly and less effective)

### Sensitivity analyses

Six sensitivity analyses were performed. Outcomes of the sensitivity analyses demonstrated many similarities with those of the main analysis (Table 3). Except for SA6, differences in QALYs were in favour of the usual care group. Cost per category for the non-operatively and operatively treated subgroup are reported separately (Additional file 2 and Additional file 3). Except for SA5, OMAS scores were higher in the reduced imaging group than in the usual care group and except for SA4 and SA5, total costs were highest in the reduced imaging group. However, all of these differences in costs and effects were not statistically significant. It is perhaps important to note that the relatively large differences in ICERs between the main analysis and some of the sensitivity analyses were due to small between-group differences in QALYs and OMAS scores.

## Discussion

The reduced imaging follow-up protocol resulted in a significant decrease in the number of radiographs as well as the associated cost compared to usual care. Other cost categories, including total healthcare costs and total societal costs, did not statistically differ between groups. Furthermore, no statistically significant differences were found between groups for QALYs and OMAS. This indicates that functional outcome and HR-QOL were unaffected by reducing the number of radiographs performed at 6 and 12 weeks follow-up. The probability of the reduced imaging protocol being cost-effective compared with usual care was relatively low (0.45) at a willingness-to-pay threshold of €20,000 per QALY. In the Netherlands, this is deemed an acceptable cost-per-QALY for interventions for diseases/disorders with a relatively low disease burden [36]. For OMAS, it is currently unknown how much decision-makers are willing to pay per unit of effect gained, so it is not possible to draw any firm conclusions for this outcome. Sensitivity analyses confirm these findings. Literature on the (cost-)effectiveness of omitting routine extremity radiography is scarce. This is discussed in our retrospective review [37] and has been confirmed by researchers investigating the usefulness of an additional shoulder radiograph [38]. Results from this study however were consistent with results from our study which examined the cost-effectiveness of reduced imaging in distal radius fractures [39]. In that study we also saw no difference in functional outcome, but a significant reduction in cost for radiographic imaging in the reduced-imaging group.

### Strengths and limitations

This economic evaluation was performed alongside a pragmatic randomised controlled trial. Therefore, our results are likely to have a high internal validity, while their external validity is improved by the pragmatic nature of the trial. Of course, this study has limitations as do all studies. First, the sample size calculation was based upon a margin of non-inferiority [40] for the OMAS, rather than a meaningful difference in societal costs or QALYs. Wide confidence intervals surrounding the aggregate and disaggregate cost differences suggest that the study was underpowered to detect a meaningful difference in cost between groups. This is common in economic evaluations as powering to detect a meaningful difference in societal costs would have required many more participants. This would have been neither feasible nor ethical. Second, the number of radiographs omitted was lower than anticipated. This was due to a high number of protocol violations in the reduced imaging group. The protocol was adhered (i.e. no routine radiograph obtained at both 6 as well as 12 weeks of follow-up) to in just 59 of 118 participants (50%) in this group. We have reported on this in more detail in an earlier report [41]. Third, self-reported questionnaires were used to query the effect, and some costs. These questionnaires had a maximum recall period of 26 weeks, which might have introduced recall bias. However, as the recall period was similar in both groups we assume that if present, this bias was similar for both groups. Fourth, 79% (195/246) of the participants had at least one missing item on at least one of the questionnaires. The number of participants with complete cost and effect data was 242 at baseline (100%), 227 at week 6 (92%), 216 at week 12 (88%), 206 at week 26 (84%), and 201 at week 52 (82%). Multiple imputation was used to deal with missing data. In an economic evaluation, multiple imputation is considered the gold-standard for dealing with missing data [32]. Moreover, a sensitivity analysis using data of complete cases showed similar results as the main analysis, i.e. no significant differences between groups for costs, OMAS and QALYs. Finally, the patients’ EQ-5D-3L health status following the fracture was not assessed, but evaluated prior to the fracture and at the various follow-up measurement points. To deal with this issue, we assumed the patients’ 6-week EQ-5D-3L health state to be representative for the complete period between the occurrence of the fracture and 6-week follow-up and used this value for calculating QALYs. We opted for this strategy, instead of using their pre-injury EQ-5D-3L health state, since most patients would have had a cast, or non-weightbearing mobilisation during these 6 weeks. We do not expect this to have biased our outcomes, since a sensitivity analysis using the patients’ EQ-5D-3L health state before the occurrence of the fracture showed similar results as the main analysis.

## Conclusion

Reducing the number of routine follow-up radiographs (on average one per patient) has a relatively low probability of being cost-effective compared with usual care. However, functional outcome, health-related quality of life and societal costs were comparable in both groups whereas imaging costs were lower in the reduced imaging group. In the light of these findings and the potential for further reduction of the number of routine follow-up radiographs in daily clinical ankle fracture care, we advise a reduced imaging follow-up protocol for patients with ankle fracture.

## Supplementary information

**Additional file 1.** Outcome scores per treatment allocation per timepoint.

**Additional file 2.** Mean cost (in euros) per conservatively treated participant in the intervention and control group and mean cost differences between groups during follow-up.

**Additional file 3.** Mean cost (in euros) per operatively treated participant in the intervention and control group and mean cost differences between groups during follow-up.

## Data Availability

The datasets analysed in the current study can be made available by the corresponding author upon request.

## Abbreviations

BMI: Body Mass Index
CEACs: Cost-effectiveness acceptability curves
CHEERS: Consolidated Health Economic Evaluation Reporting Standards
HR-QOL: Health-Related Quality Of Life
ICER: Incremental cost-effectiveness ratio
IQR: Inter-Quartile Range
NRS: Numerical Rating Scale
OMAS: Olerud&Molander Ankle Score
PA: Pronation Abduction
PE: Pronation External rotation
ProMISe: Project Manager Internet Server
QALYs: Quality-Adjusted Life Years
ROM: Range Of Motion
SA: Supination Adduction
SE: Supination External rotation
SUR: Seemingly unrelated regression analyses
95% CI: 95% confidence intervals
ΔC: Cost difference
ΔE: Effect difference
