# Supplementary material for: Reduction of routine use of radiography in patients with ankle fractures leads to lower costs and has no impact on clinical outcome: an economic evaluation
Source: BMC Health Serv Res. 2020 Sep 22;20:893. doi: 10.1186/s12913-020-05725-1 (PMC7507707; doi:10.1186/s12913-020-05725-1)
Supplement: Supplementary file 1 — Additional file 1. Outcome scores per treatment allocation per timepoint. [file 12913_2020_5725_MOESM1_ESM.docx]

|  |  | Usual care *n=128* *median (IQR)* | Reduced imaging  *n=118* *mean (IQR)* |
| --- | --- | --- | --- |
| OMAS *0-100* | *BL* | 100 (100-100) | 100 (100-100) |
|  | *W6* | 40 (25-60) | 45 (25-65) |
|  | *W12* | 65 (45-80) | 65 (46-80) |
|  | *W26* | 85 (68-95) | 80 (65-95) |
|  | *W52* | 90 (80-100) | 90 (80-100) |
|  |  |  |  |
| EQ-5D 0-1 | *BL* | 1.0 (0.9-1.0) | 1.0 (0.84-1.0) |
|  | *W6* | 0.78 (0.57-0.81) | 0.78 (0.65-0.86) |
|  | *W12* | 0.83 (0.78-1.0) | 0.81 (0.78-1.0) |
|  | *W26* | 1.0 (0.81-1.0) | 0.84 (0.78-1.0) |
|  | *W52* | 1.0 (0.84-1.0) | 1.0 (0.81-1.0) |

***Appendix C: outcome scores per treatment allocation per timepoint.***
