# Supplementary material for: Reduction of routine use of radiography in patients with ankle fractures leads to lower costs and has no impact on clinical outcome: an economic evaluation
Source: BMC Health Serv Res. 2020 Sep 22;20:893. doi: 10.1186/s12913-020-05725-1 (PMC7507707; doi:10.1186/s12913-020-05725-1)
Supplement: Supplementary file 2 — Additional file 2. Mean cost (in euros) per conservatively treated participant in the intervention and control group and mean cost differences between groups during follow-up. [file 12913_2020_5725_MOESM2_ESM.docx]

| **Cost category** | **Control n=128, mean (SEM)** | **Intervention n=118,  mean (SEM)** | **Cost difference adjusted, mean (95%CI)** |
| --- | --- | --- | --- |
| Intervention | 271 (17) | 205 (13) | **-54 (-96 to -16)** |
| Primary care | 752 (259) | 708 (177) | 63 (-448 to 564) |
| Secondary care | 3465 (1936) | 1169 (272) | -1194 (-5891 to 574) |
| Medication | 37 (14) | 18 (7) | -13 (-39 to 6) |
| Informal care | 557 (203) | 328 (135) | -161 (-728 to 145) |
| Absenteeism | 1058 (384) | 1006 (510) | -42 (-1119 to 1213) |
| Presenteeism | 4076 (949) | 3217 (957) | -405 (-2838 to 1613) |
| Unpaid productivity loss | 846 (278) | 214 (88) | **-619 (-1524 to -175)** |
| **Total** | **11063 (2665)** | **6865 (1267)** | **-2425** **(-9471 to 1162)** |

***Table 2 Mean cost (in euros) per conservatively treated participant in the intervention and control group and mean cost differences between groups during follow-up***
