# Supplementary material for: Reduction of routine use of radiography in patients with ankle fractures leads to lower costs and has no impact on clinical outcome: an economic evaluation
Source: BMC Health Serv Res. 2020 Sep 22;20:893. doi: 10.1186/s12913-020-05725-1 (PMC7507707; doi:10.1186/s12913-020-05725-1)
Supplement: Supplementary file 3 — Additional file 3. Mean cost (in euros) per operatively treated participant in the intervention and control group and mean cost differences between groups during follow-up. [file 12913_2020_5725_MOESM3_ESM.docx]

| **Cost category** | **Control n=128, mean (SEM)** | **Intervention n=118,  mean (SEM)** | **Cost difference adjusted, mean (95%CI)** |
| --- | --- | --- | --- |
| Intervention | 264 (11) | 231 (12) | **-40 (-71 to -11)** |
| Primary care | 1110 (161) | 1575 (589) | 101 (-476 to 1470) |
| Secondary care | 10064 (908) | 11469 (1679) | 249 (-2733 to 3775) |
| Medication | 35 (10) | 32 (11) | -5 (-33 to 26) |
| Informal care | 747 (143) | 824 (202) | 33 (-384 to 516) |
| Absenteeism | 923 (256) | 1335 (443) | 551 (-335 to 1746) |
| Presenteeism | 5451 (794) | 5012 (885) | 257 (-1618 to 2301) |
| Unpaid productivity loss | 753 (175) | 1058 (284) | 287 (-278 to 980) |
| **Total** | **19346 (1330)** | **21536 (2420)** | **1432** **(-2596 to 6998)** |

***Table 2 Mean cost (in euros) per operatively treated participant in the intervention and control group and mean cost differences between groups during follow-up***
